# 9-{4-[(*E*)-2-(4,6-Dimethyl-1,3,5-triazin-2-yl)ethen­yl]phen­yl}-9*H*-carbazole

**DOI:** 10.1107/S1600536809030359

**Published:** 2009-08-08

**Authors:** Chang-Lin Liu, Gang Xue, Yue-Zhi Cui, Tian-Duo Li, Seik Weng Ng

**Affiliations:** a Department of Chemical Engineering, Shandong Institute of Light Industry, Jinan 250353, Shandong Province, People’s Republic of China; b School of Materials Science and Engineering, Institute of Power Source and Ecomaterials Science, Hebei University of Technology, Tianjin 300130, People’s Republic of China; c Department of Chemistry, University of Malaya, 50603 Kuala Lumpur, Malaysia

## Abstract

In the crystal structure of the title compound, C_25_H_20_N_4_, the triazinyl ring is nearly coplanar with the planar (r.m.s. deviation = 0.028 Å) phenyl­ethenyl unit, the twist being only 5.8 (2)°; however, the planar carbazolyl unit (r.m.s. deviation = 0.008 Å) is twisted by 47.8 (1)° with respect to the phenyl­ethenyl unit. The nonplanar nature of the mol­ecule explains the phenomenon of light emission at short wavelengths in the solid state but at long wavelengths in solution.

## Related literature

For background literature on donor–π-acceptor chromophores, see: Cui *et al.* (2003 ▶, 2004 ▶); Kannan *et al.* (2004 ▶); Maury & Bozec (2005 ▶); Zhong *et al.* (2008 ▶).
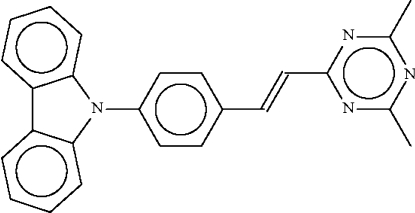


## Experimental

### 

#### Crystal data

C_25_H_20_N_4_
*M* *_r_* = 376.45
Orthorhombic, 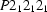

*a* = 8.0415 (8) Å
*b* = 15.716 (2) Å
*c* = 16.098 (1) Å
*V* = 2034.4 (3) Å^3^
*Z* = 4
Mo *K*α radiation
μ = 0.07 mm^−1^
*T* = 293 K
0.42 × 0.28 × 0.16 mm

#### Data collection

Siemens P4 four-circle diffractometer
Absorption correction: ψ scan (North *et al.*, 1968 ▶) *T*_min_ = 0.901, *T*_max_ = 0.988
2991 measured reflections
2291 independent reflections
1288 reflections with *I* > 2σ(*I*)
*R*_int_ = 0.026
3 standard reflections every 97 reflections intensity decay: 1%

#### Refinement

*R*[*F* ^2^ > 2σ(*F* ^2^)] = 0.055
*wR*(*F* ^2^) = 0.160
*S* = 1.00
2291 reflections
265 parameters
H-atom parameters constrained
Δρ_max_ = 0.26 e Å^−3^
Δρ_min_ = −0.30 e Å^−3^

### 

Data collection: *XSCANS* (Siemens, 1996 ▶); cell refinement: *XSCANS*; data reduction: *XSCANS*; program(s) used to solve structure: *SHELXS97* (Sheldrick, 2008 ▶); program(s) used to refine structure: *SHELXL97* (Sheldrick, 2008 ▶); molecular graphics: *X-SEED* (Barbour, 2001 ▶); software used to prepare material for publication: *publCIF* (Westrip, 2009 ▶).

## Supplementary Material

Crystal structure: contains datablocks global, I. DOI: 10.1107/S1600536809030359/bt5017sup1.cif

Structure factors: contains datablocks I. DOI: 10.1107/S1600536809030359/bt5017Isup2.hkl

Additional supplementary materials:  crystallographic information; 3D view; checkCIF report

## Figures and Tables

**Fig. 1.**
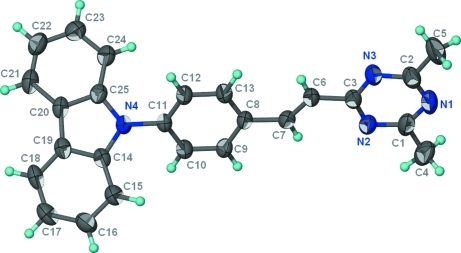
Anisotropic displacement ellipsoid plot (Barbour, 2001) of C25H20N4 with displacement ellipsoids are drawn at the 50% probability level.

**Fig. 2.**
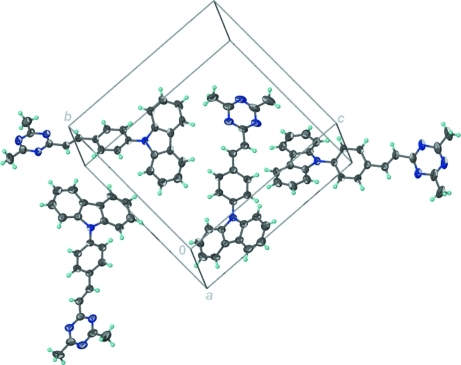
Anisotropic displacement ellipsoid plot (Barbour, 2001) depicting the alignment of four molecules in the unit cell.

## supplementary crystallographic information

### Comment

s-Triazine, which has a conjugated structure is commonly derivatized in the design of chromophores that display specific physical properties (Cui *et al.*, 2003; Maury *et al.*, 2005; Zhong *et al.*, 2008), particularly two-photon absorption (Cui *et al.*, 2004; Kannan *et al.*, 2004). The title compound (Fig. 1, Scheme 1) exemplifies a donor-π-acceptor compound with a carbazolyl donor and an *s*-triazinyl acceptor. It emits blue light in solid state and yellow-green light in solution. However, this property is unusual as most compounds show a bathochromic shift of fluorescence in solid state relative to their emission in solution.

An intramolecular charge transfer (ICT) axis runs from atom N2 to atom N4. The phenylethenyl unit is almost coplanar with the triazinyl ring. However, the carbazolyl unit is severely twisted with respect to the phenylethenyl and triazinyl units, so that conjugation is poor. Accordingly, such a poorly-conjugated molecule can only emit at a short wavelength (in the solid state) whereas in solution, the molecule is probably freed from strain and can achieve planarity. Consequently, it emits at longer wavelengths. The molecules are packed such that the axes of one half the number molecules are aligned in one direction whereas those of the other half are aligned approximately perpendicular to it (Fig. 2).

### Experimental

4-*N*-Carbazolylbenzaldehyde (2.03 g, 7.5 mmol) in methanol (30 ml) was added to 2,4,6-trimethyl-*s*-triazine (1.85 g, 15 mmol) and potassium hydroxide (0.5 g) in methanol (50 ml). The mixture was heated for 24 h. The solvent was removed and the residue was purified by column chromatography on silica gel by using benzene/ethanol (10/1) as eluent. Crystals were obtained by recrystallization from a benzene/ethanol solution of the compound.

### Refinement

Due to the absence of anomalous scatterers, 1509 Friedel pairs were merged. Hydrogen atoms were geometrically fixed and allowed to ride on their parent atoms, with C–H 0.93–0.96 Å and *U*_iso_(H) set to 1.2–1.5*U*_eq_(C).

### Crystal data

**Table:**

C_25_H_20_N_4_	*F*(000) = 792
*M**_r_* = 376.45	*D*_x_ = 1.229 Mg m^−^^3^
Orthorhombic, *P*2_1_2_1_2_1_	Mo *K*α radiation, λ = 0.71073 Å
Hall symbol: P 2ac 2ab	Cell parameters from 40 reflections
*a* = 8.0415 (8) Å	θ = 4.6–12.4°
*b* = 15.716 (2) Å	µ = 0.07 mm^−^^1^
*c* = 16.098 (1) Å	*T* = 293 K
*V* = 2034.4 (3) Å^3^	Prism, pale green
*Z* = 4	0.42 × 0.28 × 0.16 mm

### Data collection

**Table:**

Siemens P4 four-circle diffractometer	1288 reflections with *I* > 2σ(*I*)
Radiation source: fine-focus sealed tube	*R*_int_ = 0.026
graphite	θ_max_ = 26.0°, θ_min_ = 1.8°
ω scans	*h* = −1→9
Absorption correction: ψ scan (North *et al.*, 1968)	*k* = −19→1
*T*_min_ = 0.901, *T*_max_ = 0.988	*l* = −19→1
2991 measured reflections	3 standard reflections every 97 reflections
2291 independent reflections	intensity decay: 1%

### Refinement

**Table:**

Refinement on *F*^2^	Secondary atom site location: difference Fourier map
Least-squares matrix: full	Hydrogen site location: inferred from neighbouring sites
*R*[*F*^2^ > 2σ(*F*^2^)] = 0.055	H-atom parameters constrained
*wR*(*F*^2^) = 0.160	*w* = 1/[σ^2^(*F*_o_^2^) + (0.0785*P*)^2^] where *P* = (*F*_o_^2^ + 2*F*_c_^2^)/3
*S* = 1.00	(Δ/σ)_max_ = 0.001
2291 reflections	Δρ_max_ = 0.26 e Å^−^^3^
265 parameters	Δρ_min_ = −0.30 e Å^−^^3^
0 restraints	Extinction correction: *SHELXL97* (Sheldrick, 2008), Fc^*^=kFc[1+0.001xFc^2^λ^3^/sin(2θ)]^-1/4^
Primary atom site location: structure-invariant direct methods	Extinction coefficient: 0.063 (5)

### Fractional atomic coordinates and isotropic or equivalent isotropic displacement parameters (Å^2^)

**Table:**

	*x*	*y*	*z*	*U*_iso_*/*U*_eq_	
N1	0.3440 (7)	0.5461 (3)	0.7650 (3)	0.0774 (15)	
N2	0.2899 (6)	0.4809 (2)	0.6348 (2)	0.0627 (12)	
N3	0.4255 (6)	0.4024 (3)	0.7419 (3)	0.0659 (12)	
N4	0.3597 (5)	0.0613 (2)	0.3022 (2)	0.0511 (11)	
C1	0.2848 (8)	0.5466 (3)	0.6880 (4)	0.0732 (17)	
C2	0.4126 (8)	0.4724 (4)	0.7893 (3)	0.0727 (17)	
C3	0.3621 (7)	0.4108 (3)	0.6658 (3)	0.0584 (14)	
C4	0.2098 (9)	0.6278 (3)	0.6563 (4)	0.091 (2)	
H4A	0.1203	0.6448	0.6921	0.137*	
H4B	0.1680	0.6190	0.6011	0.137*	
H4C	0.2932	0.6715	0.6554	0.137*	
C5	0.4801 (8)	0.4663 (4)	0.8757 (3)	0.097 (2)	
H5A	0.4946	0.4076	0.8903	0.146*	
H5B	0.4036	0.4925	0.9137	0.146*	
H5C	0.5853	0.4950	0.8785	0.146*	
C6	0.3767 (7)	0.3359 (3)	0.6121 (3)	0.0563 (13)	
H6	0.4272	0.2875	0.6337	0.068*	
C7	0.3217 (7)	0.3333 (3)	0.5341 (3)	0.0539 (12)	
H7	0.2704	0.3823	0.5144	0.065*	
C8	0.3331 (7)	0.2616 (3)	0.4757 (3)	0.0498 (12)	
C9	0.2847 (7)	0.2736 (3)	0.3941 (3)	0.0545 (13)	
H9	0.2475	0.3269	0.3771	0.065*	
C10	0.2910 (7)	0.2076 (3)	0.3373 (3)	0.0544 (13)	
H10	0.2559	0.2166	0.2830	0.065*	
C11	0.3490 (6)	0.1284 (3)	0.3607 (3)	0.0471 (12)	
C12	0.3989 (7)	0.1159 (3)	0.4417 (3)	0.0537 (12)	
H12	0.4389	0.0630	0.4581	0.064*	
C13	0.3898 (7)	0.1816 (3)	0.4988 (3)	0.0563 (13)	
H13	0.4223	0.1720	0.5534	0.068*	
C14	0.4250 (6)	0.0677 (3)	0.2221 (3)	0.0503 (12)	
C15	0.5018 (7)	0.1357 (3)	0.1836 (3)	0.0630 (14)	
H15	0.5147	0.1875	0.2108	0.076*	
C16	0.5584 (7)	0.1243 (4)	0.1036 (3)	0.0700 (15)	
H16	0.6104	0.1693	0.0766	0.084*	
C17	0.5401 (7)	0.0476 (4)	0.0625 (3)	0.0712 (16)	
H17	0.5785	0.0419	0.0083	0.085*	
C18	0.4658 (6)	−0.0198 (4)	0.1013 (3)	0.0636 (15)	
H18	0.4539	−0.0713	0.0735	0.076*	
C19	0.4080 (6)	−0.0115 (3)	0.1823 (3)	0.0521 (12)	
C20	0.3274 (6)	−0.0680 (3)	0.2402 (3)	0.0520 (12)	
C21	0.2764 (7)	−0.1526 (3)	0.2366 (3)	0.0662 (15)	
H21	0.2946	−0.1844	0.1888	0.079*	
C22	0.2003 (8)	−0.1884 (3)	0.3032 (3)	0.0733 (16)	
H22	0.1661	−0.2448	0.3006	0.088*	
C23	0.1725 (7)	−0.1418 (3)	0.3754 (3)	0.0688 (15)	
H23	0.1202	−0.1675	0.4204	0.083*	
C24	0.2220 (7)	−0.0573 (3)	0.3811 (3)	0.0624 (14)	
H24	0.2028	−0.0260	0.4291	0.075*	
C25	0.3004 (6)	−0.0213 (3)	0.3137 (3)	0.0520 (12)	

### Atomic displacement parameters (Å^2^)

**Table:**

	*U*^11^	*U*^22^	*U*^33^	*U*^12^	*U*^13^	*U*^23^
N1	0.088 (4)	0.069 (3)	0.075 (3)	−0.020 (3)	0.025 (3)	−0.033 (3)
N2	0.075 (3)	0.049 (2)	0.065 (2)	−0.009 (2)	0.021 (3)	−0.010 (2)
N3	0.077 (3)	0.064 (3)	0.056 (2)	−0.019 (3)	0.017 (3)	−0.019 (2)
N4	0.064 (3)	0.044 (2)	0.045 (2)	−0.002 (2)	0.001 (2)	−0.0078 (17)
C1	0.081 (4)	0.057 (3)	0.081 (4)	−0.021 (3)	0.031 (4)	−0.025 (3)
C2	0.075 (4)	0.083 (4)	0.060 (3)	−0.030 (4)	0.023 (3)	−0.023 (3)
C3	0.067 (4)	0.055 (3)	0.053 (3)	−0.015 (3)	0.017 (3)	−0.013 (2)
C4	0.108 (5)	0.053 (3)	0.114 (5)	0.003 (3)	0.038 (5)	−0.016 (3)
C5	0.099 (5)	0.126 (5)	0.066 (4)	−0.037 (4)	0.013 (4)	−0.041 (4)
C6	0.072 (4)	0.047 (3)	0.050 (3)	−0.008 (3)	0.011 (3)	−0.009 (2)
C7	0.065 (3)	0.043 (2)	0.053 (3)	−0.005 (3)	0.008 (3)	−0.004 (2)
C8	0.060 (3)	0.041 (2)	0.049 (3)	−0.005 (2)	0.004 (3)	−0.007 (2)
C9	0.071 (4)	0.040 (2)	0.052 (3)	0.006 (3)	0.001 (3)	−0.005 (2)
C10	0.071 (3)	0.047 (3)	0.046 (2)	0.004 (3)	−0.002 (3)	−0.005 (2)
C11	0.056 (3)	0.043 (2)	0.043 (2)	0.000 (2)	0.001 (2)	−0.008 (2)
C12	0.071 (3)	0.042 (2)	0.048 (3)	0.004 (3)	−0.006 (3)	−0.006 (2)
C13	0.074 (4)	0.051 (3)	0.043 (2)	−0.001 (3)	−0.004 (3)	−0.007 (2)
C14	0.052 (3)	0.054 (3)	0.044 (3)	0.006 (3)	0.000 (2)	−0.006 (2)
C15	0.074 (4)	0.063 (3)	0.051 (3)	−0.003 (3)	0.002 (3)	−0.006 (3)
C16	0.077 (4)	0.084 (4)	0.049 (3)	−0.005 (4)	0.007 (3)	−0.001 (3)
C17	0.074 (4)	0.094 (4)	0.045 (3)	0.007 (4)	0.003 (3)	−0.006 (3)
C18	0.064 (4)	0.076 (3)	0.050 (3)	0.011 (3)	−0.006 (3)	−0.026 (3)
C19	0.051 (3)	0.057 (3)	0.048 (3)	0.008 (3)	−0.007 (2)	−0.010 (2)
C20	0.053 (3)	0.047 (3)	0.056 (3)	0.006 (2)	−0.009 (3)	−0.012 (2)
C21	0.072 (4)	0.054 (3)	0.072 (3)	0.004 (3)	−0.011 (3)	−0.019 (3)
C22	0.091 (4)	0.047 (3)	0.082 (4)	−0.001 (3)	−0.005 (4)	−0.007 (3)
C23	0.078 (4)	0.057 (3)	0.072 (3)	−0.004 (3)	0.008 (3)	0.003 (3)
C24	0.073 (4)	0.049 (3)	0.065 (3)	0.003 (3)	0.007 (3)	−0.008 (2)
C25	0.057 (3)	0.044 (2)	0.055 (3)	0.004 (2)	−0.005 (3)	−0.004 (2)

### Geometric parameters (Å, °)

**Table:**

N1—C1	1.328 (7)	C10—H10	0.9300
N1—C2	1.342 (7)	C11—C12	1.378 (6)
N2—C1	1.341 (5)	C12—C13	1.384 (6)
N2—C3	1.341 (6)	C12—H12	0.9300
N3—C3	1.333 (6)	C13—H13	0.9300
N3—C2	1.342 (6)	C14—C15	1.382 (6)
N4—C25	1.396 (6)	C14—C19	1.406 (6)
N4—C14	1.396 (5)	C15—C16	1.378 (6)
N4—C11	1.416 (5)	C15—H15	0.9300
C1—C4	1.500 (7)	C16—C17	1.382 (7)
C2—C5	1.496 (8)	C16—H16	0.9300
C3—C6	1.465 (6)	C17—C18	1.367 (7)
C4—H4A	0.9600	C17—H17	0.9300
C4—H4B	0.9600	C18—C19	1.391 (6)
C4—H4C	0.9600	C18—H18	0.9300
C5—H5A	0.9600	C19—C20	1.441 (6)
C5—H5B	0.9600	C20—C21	1.393 (6)
C5—H5C	0.9600	C20—C25	1.408 (6)
C6—C7	1.332 (6)	C21—C22	1.357 (7)
C6—H6	0.9300	C21—H21	0.9300
C7—C8	1.471 (5)	C22—C23	1.392 (7)
C7—H7	0.9300	C22—H22	0.9300
C8—C9	1.383 (6)	C23—C24	1.389 (6)
C8—C13	1.388 (6)	C23—H23	0.9300
C9—C10	1.383 (6)	C24—C25	1.377 (6)
C9—H9	0.9300	C24—H24	0.9300
C10—C11	1.382 (6)		
			
C1—N1—C2	115.1 (4)	C10—C11—N4	120.6 (4)
C1—N2—C3	114.1 (4)	C11—C12—C13	120.4 (4)
C3—N3—C2	114.3 (5)	C11—C12—H12	119.8
C25—N4—C14	108.5 (4)	C13—C12—H12	119.8
C25—N4—C11	125.8 (4)	C12—C13—C8	121.0 (4)
C14—N4—C11	125.7 (4)	C12—C13—H13	119.5
N1—C1—N2	125.4 (5)	C8—C13—H13	119.5
N1—C1—C4	117.8 (5)	C15—C14—N4	129.6 (4)
N2—C1—C4	116.7 (5)	C15—C14—C19	121.6 (4)
N3—C2—N1	125.0 (5)	N4—C14—C19	108.8 (4)
N3—C2—C5	116.6 (6)	C16—C15—C14	117.8 (5)
N1—C2—C5	118.3 (5)	C16—C15—H15	121.1
N3—C3—N2	126.1 (4)	C14—C15—H15	121.1
N3—C3—C6	115.6 (5)	C15—C16—C17	121.8 (5)
N2—C3—C6	118.4 (4)	C15—C16—H16	119.1
C1—C4—H4A	109.5	C17—C16—H16	119.1
C1—C4—H4B	109.5	C18—C17—C16	120.2 (5)
H4A—C4—H4B	109.5	C18—C17—H17	119.9
C1—C4—H4C	109.5	C16—C17—H17	119.9
H4A—C4—H4C	109.5	C17—C18—C19	120.1 (5)
H4B—C4—H4C	109.5	C17—C18—H18	120.0
C2—C5—H5A	109.5	C19—C18—H18	120.0
C2—C5—H5B	109.5	C18—C19—C14	118.6 (5)
H5A—C5—H5B	109.5	C18—C19—C20	134.3 (5)
C2—C5—H5C	109.5	C14—C19—C20	107.1 (4)
H5A—C5—H5C	109.5	C21—C20—C25	119.1 (5)
H5B—C5—H5C	109.5	C21—C20—C19	133.9 (5)
C7—C6—C3	123.6 (5)	C25—C20—C19	107.0 (4)
C7—C6—H6	118.2	C22—C21—C20	119.7 (5)
C3—C6—H6	118.2	C22—C21—H21	120.1
C6—C7—C8	127.3 (5)	C20—C21—H21	120.1
C6—C7—H7	116.4	C21—C22—C23	121.0 (5)
C8—C7—H7	116.4	C21—C22—H22	119.5
C9—C8—C13	118.1 (4)	C23—C22—H22	119.5
C9—C8—C7	119.0 (4)	C24—C23—C22	120.8 (5)
C13—C8—C7	122.9 (4)	C24—C23—H23	119.6
C8—C9—C10	121.0 (4)	C22—C23—H23	119.6
C8—C9—H9	119.5	C25—C24—C23	118.2 (5)
C10—C9—H9	119.5	C25—C24—H24	120.9
C9—C10—C11	120.5 (4)	C23—C24—H24	120.9
C9—C10—H10	119.8	C24—C25—N4	130.0 (4)
C11—C10—H10	119.8	C24—C25—C20	121.2 (4)
C12—C11—C10	119.0 (4)	N4—C25—C20	108.7 (4)
C12—C11—N4	120.3 (4)		
			
C2—N1—C1—N2	−0.5 (9)	C25—N4—C14—C19	−0.3 (5)
C2—N1—C1—C4	−178.7 (5)	C11—N4—C14—C19	−177.8 (4)
C3—N2—C1—N1	0.1 (8)	N4—C14—C15—C16	178.7 (5)
C3—N2—C1—C4	178.3 (5)	C19—C14—C15—C16	1.4 (8)
C3—N3—C2—N1	−0.4 (8)	C14—C15—C16—C17	0.0 (8)
C3—N3—C2—C5	179.4 (5)	C15—C16—C17—C18	−0.7 (9)
C1—N1—C2—N3	0.7 (9)	C16—C17—C18—C19	0.1 (8)
C1—N1—C2—C5	−179.2 (5)	C17—C18—C19—C14	1.2 (7)
C2—N3—C3—N2	0.0 (8)	C17—C18—C19—C20	−179.4 (5)
C2—N3—C3—C6	179.0 (4)	C15—C14—C19—C18	−1.9 (7)
C1—N2—C3—N3	0.1 (8)	N4—C14—C19—C18	−179.8 (4)
C1—N2—C3—C6	−178.9 (5)	C15—C14—C19—C20	178.5 (5)
N3—C3—C6—C7	−179.4 (5)	N4—C14—C19—C20	0.6 (5)
N2—C3—C6—C7	−0.3 (8)	C18—C19—C20—C21	−0.2 (10)
C3—C6—C7—C8	179.2 (5)	C14—C19—C20—C21	179.3 (5)
C6—C7—C8—C9	−173.2 (5)	C18—C19—C20—C25	179.7 (5)
C6—C7—C8—C13	7.1 (8)	C14—C19—C20—C25	−0.8 (5)
C13—C8—C9—C10	0.7 (8)	C25—C20—C21—C22	0.7 (8)
C7—C8—C9—C10	−179.1 (5)	C19—C20—C21—C22	−179.3 (6)
C8—C9—C10—C11	−1.3 (8)	C20—C21—C22—C23	−0.2 (9)
C9—C10—C11—C12	0.7 (8)	C21—C22—C23—C24	0.1 (9)
C9—C10—C11—N4	−178.3 (5)	C22—C23—C24—C25	−0.5 (8)
C25—N4—C11—C12	49.7 (7)	C23—C24—C25—N4	178.9 (5)
C14—N4—C11—C12	−133.2 (5)	C23—C24—C25—C20	1.1 (8)
C25—N4—C11—C10	−131.3 (5)	C14—N4—C25—C24	−178.2 (5)
C14—N4—C11—C10	45.8 (7)	C11—N4—C25—C24	−0.7 (8)
C10—C11—C12—C13	0.5 (8)	C14—N4—C25—C20	−0.2 (5)
N4—C11—C12—C13	179.5 (5)	C11—N4—C25—C20	177.3 (4)
C11—C12—C13—C8	−1.0 (8)	C21—C20—C25—C24	−1.2 (7)
C9—C8—C13—C12	0.4 (8)	C19—C20—C25—C24	178.8 (4)
C7—C8—C13—C12	−179.8 (5)	C21—C20—C25—N4	−179.4 (5)
C25—N4—C14—C15	−177.9 (5)	C19—C20—C25—N4	0.6 (5)
C11—N4—C14—C15	4.6 (8)		
